# Low 1,25-dihydroxyvitamin D levels are associated with severe COVID-19: an observational study in hospitalized patients

**DOI:** 10.3389/fnut.2026.1836021

**Published:** 2026-06-05

**Authors:** Esra Canbolat Unlu, Semsi Nur Karabela, Zuhal Yesilbag, Ramazan Korkusuz, Deniz Borcak, Esra Salim Dogdas, Sevtap Senoglu, Nilgun Isiksacan, Kadriye Kart Yasar

**Affiliations:** 1 Department of Infectious Diseases and Clinical Microbiology, Bakirkoy Dr. Sadi Konuk Training and Research Hospital, University of Health Sciences, Istanbul, Türkiye; 2 Department of Clinical Biochemistry, Bakirkoy Dr. Sadi Konuk Training and Research Hospital, University of Health Sciences, Istanbul, Türkiye

**Keywords:** 1,25-dihydroxyvitamin D, 25-hydroxyvitamin D, COVID-19 severity, hospitalized patients, prognostic biomarker, vitamin D deficiency

## Abstract

**Background:**

This study aimed to evaluate the association of serum 25-hydroxyvitamin D (25(OH)D) and 1,25-dihydroxyvitamin D (1,25(OH)_2_D) levels with clinical severity and mortality among hospitalized patients with moderate and severe COVID-19.

**Methods:**

A total of 185 adult patients (≥18 years) with confirmed SARS-CoV-2 PCR positivity and without chronic kidney disease, admitted between March and May 2020, were included. Clinical characteristics, laboratory parameters, and serum 25(OH)D and 1,25(OH)_2_D levels were assessed. Factors associated with severe disease and mortality were determined using multivariate logistic regression and Cox regression analyses.

**Results:**

Of the patients, 114 had moderate disease and 71 had severe disease. Length of hospital stay, intensive care unit (ICU) admission, and mortality rates were significantly higher in the severe group. While 25(OH)D levels were lower in the moderate group, 1,25(OH)_2_D levels were markedly reduced in severe cases. Multivariate analysis identified prolonged hospital stay, ICU admission history, and low 1,25(OH)_2_D levels were independently associated with severe disease. Independently associated factors with mortality were ICU admission (*p* < 0.001), high troponin levels (*p* = 0.003) and prolonged activated partial thromboplastin time (APTT) (*p* = 0.04). Although vitamin D deficiency was associated with severe disease, it was not independently associated with mortality.

**Conclusion:**

Reduced 1,25(OH)_2_D levels are associated with severe COVID-19 and may serve as a meaningful biomarker for predicting disease severity. These findings suggest that assessing 1,25(OH)_2_D levels may support clinical decision-making, particularly in patients at risk for severe COVID-19 or ARDS.

## 1 Introduction

Coronavirus disease 2019 (COVID-19), caused by the severe acute respiratory syndrome coronavirus 2 (SARS-CoV-2), is a rapidly spreading viral infection with a clinical presentation ranging from mild symptoms to life-threatening disease. Advanced age, male sex, obesity, and cardiometabolic comorbidities have been consistently identified as major determinants of severe disease and mortality (1–3). In patients who develop critical illness, cytokine storm, acute respiratory distress syndrome (ARDS), cardiac injury, and secondary infections are key contributors to intensive care unit (ICU) admission and increased fatality.

Given its immunomodulatory properties, vitamin D has become a focus of interest in understanding factors that may influence the progression of COVID-19. Cholecalciferol synthesized in the skin from 7-dehydrocholesterol upon exposure to UVB radiation is hydroxylated in the liver to 25-hydroxyvitamin D (25(OH)D), and subsequently converted in the kidneys to the biologically active metabolite 1,25-dihydroxyvitamin D (1,25(OH)_2_D) (4). This active form plays a central role in immune regulation through modulation of T- and B-cell activity, enhancement of regulatory T-cell function, cytokine balance, and maintenance of epithelial barrier integrity (5, 6). Its ability to suppress pro-inflammatory cytokines and augment antiviral responses highlights a plausible mechanistic link with the immunopathogenesis of COVID-19 (7). Furthermore, chronic vitamin D deficiency has been associated with activation of the renin–angiotensin system, increased fibrotic signaling, and enhanced susceptibility to lung injury (8).

Despite substantial interest, findings regarding the association between vitamin D status and COVID-19 outcomes remain inconsistent. Several studies have reported no significant relationship between serum 25(OH)D levels and clinical severity or mortality (9–13), whereas numerous others have demonstrated associations between low vitamin D levels and adverse outcomes such as severe disease, ARDS, ICU requirement, and renal dysfunction (14–21). Meta-analytic evidence largely supports an association between low serum 25(OH)D concentrations and severe COVID-19 (22). Nevertheless, meta-analyses evaluating vitamin D supplementation have generally found insufficient evidence to support its protective or therapeutic efficacy (23), although some reports suggest that vitamin D administered after COVID-19 diagnosis may reduce ICU admission and mortality (24).

In contrast to 25(OH)D, data regarding the clinical relevance of the active metabolite 1,25(OH)_2_D in COVID-19 remain limited. Considering the central role of active vitamin D in immunological and inflammatory pathways, elucidating its relationship with disease severity may provide valuable insights into host response mechanisms. To our knowledge, relatively few studies have simultaneously evaluated both circulating 25(OH)D and the biologically active metabolite 1,25(OH)_2_D in hospitalized COVID-19 patients. Therefore, this study aims to evaluate the association of serum 25(OH)D and 1,25(OH)_2_D levels with clinical course, disease severity, and mortality in hospitalized patients with varying degrees of COVID-19.

## 2 Materials and methods

### 2.1 Study design and data collection

This retrospective observational cohort study included adult patients (≥18 years) who were hospitalized with confirmed SARS-CoV-2 infection by polymerase chain reaction (PCR) between March and May 2020. Patients with chronic kidney disease were excluded due to potential alterations in vitamin D metabolism. Blood samples were collected at the time of hospital admission as part of routine clinical evaluation. Serum samples were processed according to standard laboratory procedures and stored at −80 °C until analysis. Prior to measurement, samples were thawed under controlled conditions and analyzed in batches. The study protocol was approved by the Clinical Research Ethics Committee of Bakırköy Dr. Sadi Konuk Training and Research Hospital (Approval No: 2020/215).

Demographic data (age, sex), comorbidities, clinical findings, laboratory parameters, extent of pulmonary involvement, length of hospital stay, intensive care unit (ICU) requirement, and mortality outcomes were recorded for all patients. Disease severity was classified as moderate or severe/critical according to the World Health Organization (WHO) criteria. The inclusion criteria were: age ≥18 years, patients presenting with symptoms of acute respiratory tract infection, including fever, cough, or dyspnea and required hospitalization for severe acute respiratory infection, and had a positive SARS-CoV-2 PCR test result. The exclusion criteria were: age <18 years, patients with a negative or unavailable SARS-CoV-2 PCR test result, and presence of chronic kidney disease. In accordance with national COVID-19 guidelines in Türkiye, patients with mild disease were managed on an outpatient basis; therefore, only hospitalized patients with moderate or severe disease were included in the analysis.

Serum concentrations of 25-hydroxyvitamin D (25(OH)D) and 1,25-dihydroxyvitamin D (1,25(OH)_2_D) were measured using the ALL SHENG ELISA analyzer with Abbkine commercial kits, in accordance with the manufacturer’s instructions. Calibration and quality control procedures were performed using kit-provided standards and controls. Concentrations were automatically calculated by the analyzer software based on calibration curves, and no manual calculation was required. The detection range for both assays was 10–160 ng/mL. Based on established literature, vitamin D deficiency was defined as <20 ng/mL for 25(OH)D and <18 pg/mL for 1,25(OH)_2_D (25).

### 2.2 Statistical analysis

Statistical analyses were performed by using the Statistical Package for Social Sciences version 25.0 for Windows (SPSS Inc., Chicago, IL, United States). Descriptive data were presented as mean ± standard deviation, frequency, median (interquartile range), and percentage values. Categorical variables were compared using the Chi-square test and Fisher’s exact test. The normality of all continuous variables was tested with the Kolmogorov Smirnov test. The variable “mean age” was found normally distributed and Student’s *t*-test was used for comparing mean age between groups. Except the mean age, all of the continuous variables (laboratory parameters and length of hospital stay) were not normally distributed and, the Mann–Whitney *U* test was used for comparing these variables. Significant variables in the univariate analysis were included in the multivariate analysis. Logistic regression analysis and Cox-regression analysis (for mortality) were used as multivariate analysis. The “*p*”-values less than or equal to 0.05 (*p* ≤ 0.05) were considered statistically significant.

## 3 Results

A total of 185 patients were included in the study, of whom 114 had moderate disease and 71 had severe disease. The mean age was 60.03 ± 16.98 years in the moderate group (47.4% male) and 61.25 ± 16.25 years in the severe group (57.7% male). The two groups were comparable in terms of sex distribution, mean age, and underlying comorbidities.

Length of hospital stay, intensive care unit (ICU) admission, and mortality rates were significantly higher among patients with severe disease. Serum 1,25(OH)_2_D levels were significantly lower in the severe group, whereas 25(OH)D levels were lower in the moderate group.

Among laboratory findings, levels of AST (aspartate aminotransferase), urea, creatinine, LDH (lactate dehydrogenase), CRP (C-reactive protein), ferritin, procalcitonin, troponin, and PT (prothrombin time) were significantly higher in the severe group, while albumin levels and lymphocyte counts were notably lower. In multivariate analysis, prolonged hospital stay, a prior ICU admission, and reduced 1,25(OH)_2_D levels were independently associated with severe disease. The mortality rate was significantly higher in the severe group (*p* < 0.001, OR = 0.067, 95% CI: 0.01–0.30) (Table 1).

**Table 1:** Comparison of demographic, clinical and laboratory parameters of patients with moderate and severe COVID-19.

Variable	Moderate (*n* = 114)	Severe (*n* = 71)	Univariate analysis		Multivariate analysis	
			*p*-value	OR^§^ (95% CI)	*p*-value	OR^§^ (95% CI)
Sex, *n* (%)						
Male	54 (47.4)	41 (57.7)	0.170	0.65 (0.36–1.19)		
Female	60 (52.6)	30 (42.3)				
Age, years, mean ± SD	60.03 ± 16.98	61.25 ± 16.25	0.628			
Length of hospital stay, days, median (IQR)	8 (6–12)	13 (10–22)	<0.001*		0.005*	1.09 (1.02–1.15)
Prior ICU admission, *n* (%)	1 (0.9)	22 (31.0)	<0.001*	50.73 (6.65–387.01)	0.012*	15.80 (1.83–135.88)
Mortality, *n* (%)	2 (1.8)	15 (21.1)	<0.001*	15 (3.31–67.89)		
25(OH)D deficiency (<20 ng/mL), *n* (%)	78 (68.4)	19 (26.8)	<0.001*	0.169 (0.08–0.32)	<0.001*	4.42 (2.02–9.66)
1,25(OH)_2_D deficiency (<18 pg/mL), *n* (%)	75 (65.8)	63 (88.7)	<0.001*	4.095 (1.78–9.40)	0.003*	4.58 (1.67–12.53)

Comorbidities, *n* (%)	Moderate (*n* = 114)	Severe (*n* = 71)	Univariate analysis		Multivariate analysis	
			*p*-value	OR § (95% CI)	*p*-value	OR § (95% CI)
Diabetes mellitus	31 (27.4)	27 (38.0)	0.132	1.62 (0.86–3.05)		
Hypertension	61 (53.5)	31 (43.7)	0.193	0.67 (0.37–1.22)		
Coronary artery disease	35 (30.7)	14 (19.7)	0.100	0.55 (0.27–1.12)		
COPD	17 (14.9)	11 (15.5)	0.915	1.04 (0.45–2.38)		
Malignancy	6 (5.3)	6 (8.5)	0.54	1.66 (0.51–5.36)		

Laboratory findings, median (IQR)	Moderate (*n* = 114)	Severe (*n* = 71)	Univariate analysis		Multivariate analysis	
			*p*-value	OR^§^ (95% CI)	*p*-value	OR^§^ (95% CI)
Hemoglobin (g/dL)	12.5 (10.47–13.60)	11.6 (10.00–13.40)	0.105			
Hematocrit (%)	38.0 (33.0–40.80)	35.35 (31.30–40.12)	0.234			
WBC (×10^3^ μL)	7.38 (5.58–11.41)	7.93 (6.01–11.55)	0.792			
Lymphocyte (×10^3^ μL)	1.48 (1.04–1.92)	1.17 (0.85–1.62)	0.002^*^		0.587	0.892 (0.59–1.34)
Neutrophil (×10^3^ μL)	5.21 (3.33–8.72)	5.45 (4.08–8.91)	0.337			
Platelet (×10^3^ μL)	220.50 (171.00–292.50)	214.00 (143.00–299.00)	0.525			
AST (IU/L)	27.00 (20.00–41.00)	34.00 (22.00–54.00)	0.041^*^		0.264	1 (0.99–1.02)
ALT (IU/L)	21.00 (13.00–36.25)	24.00 (14.00–37.00)	0.422			
Urea (mg/dL)	33.00 (23.00–42.00)	37.00 (29.00–59.40)	0.007^*^		0.67	0.99 (0.96–1.01)
Creatinine (mg/dL)	0.79 (0.62–1.04)	0.93 (0.73–1.33)	0.007^*^		0.63	1.42 (0.33–6.14)
LDH (U/L)	255.50 (202.75–322.25)	317.00 (230.00–476.00)	<0.001^*^			
Albumin (g/L)	37.05 (33.35–40.22)	33.50 (29.50–37.70)	<0.001^*^		0.238	0.95 (0.87–1.03)
Ferritin (μg/L)	127.25 (51.17–286.67)	308.90 (123.90–672.40)	<0.001^*^		0.67	1 (0.99–1)
Triglycerides (mg/dL)	116.50 (86.00–176.25)	108.50 (79.25–172.50)	0.360			
CK (U/L)	76.50 (48.00–128.00)	92.00 (46.00–237.00)	0.124			
Procalcitonin (ng/mL)	0.07 (0.040–0.22)	0.17 (0.10–0.65)	<0.001^*^		0.257	0.91 (0.79–1.06)
CRP (mg/L)	26.00 (9.00–104.25)	90.00 (43.97–166.61)	<0.001^*^			
Fibrinogen (mg/dL)	469.50 (390.00–537.00)	494.00 (405.00–566.00)	0.066			
PT (s)	12.90 (0.00–15.27)	14.10 (12.80–15.70)	0.004^*^		0.49	1.01 (0.97–1.06)
APTT (s)	34.90 (31.47–39.42)	36.20 (32.90–38.60)	0.200			
D-dimer (mg/L)	0.42 (0.16–0.85)	0.49 (0.25–1.30)	0.146			
Troponin (ng/I)	5.00 (3.00–11.00)	10.00 (5.00–20.00)	<0.001^*^		0.74	1 (0.99–1.01)
25(OH)D (ng/mL)	15.58 (10.33–22.66)	23.50 (19.50–28.35)	<0.001^*^			
1,25(OH)_2_D (pg/mL)	16.29 (12.19–19.36)	10.70 (8.73–14.64)	<0.001^*^			

SD, standard deviation; IQR, interquartile range; OR, odds ratio; CI, confidence interval; ICU, intensive care unit; COPD, chronic obstructive pulmonary disease; WBC, White blood cell; AST, aspartate aminotransferase; ALT, alanine aminotransferase; LDH, lactate dehydrogenase; CK, creatine kinase; CRP, C-reactive protein; PT, prothrombin time; APTT, activated partial thromboplastin time. ^§^ORs are presented according to the reference categories defined in the regression model. ^*^Statistically significant values.

In the overall study population, sex and age were not associated with mortality; however, diabetes mellitus was identified as a mortality-related risk factor. Additionally, rate of severe disease and ICU admission history were found significantly higher in non-survivor group. Although vitamin D deficiency was associated with severe disease, it was not independently associated with mortality. In multivariate analysis of variables significantly associated with mortality in univariate analysis, ICU admission history (*p* < 0.001, OR = 63.98, 95% CI: 7.18–569.3), high troponin levels (*p* = 0.003, OR = 1, 95% CI: 1.003–1.005) and prolonged APTT (activated partial thromboplastin time) (*p* = 0.009, OR = 1.09, 95% CI: 1.02–1.16) were found independently associated with mortality (Table 2).

**Table 2:** Factors associated with mortality in hospitalized patients with COVID-19.

Variable	Survivors (*n* = 168)	Non-survivors (*n* = 17)	Univariate analysis		Multivariate analysis	
			*p*-value	OR^§^ (95% CI)	*p*-value	OR^§^ (95% CI)
Sex, *n* (%)						
Male	85 (50.6)	10 (58.8)	0.51	0.71 (0.26–1.97)		
Female	83 (49.4)	7 (41.2)				
Age, years, mean ± SD	60.64 ± 16.62	59.06 ± 17.63	0.71			
Length of hospital stay, days, median (IQR)	10 (7–15)	13 (8.5–25)	0.06			
Prior ICU admission, *n* (%)	8 (4.8)	15 (88.2)	<0.001*	150 (29.17–771.25)	<0.001*	63.98 (7.18–569.3)
Disease severity, *n* (%)						
Moderate	112 (66.7)	2 (11.8)	<0.001*	0.067 (0.01-0.30)	0.46	2.88 (0.16–50.14)
Severe/critical	56 (33.3)	15 (88.2)				
25(OH)D deficiency (<20 ng/mL), *n* (%)	95 (56.5)	2 (11.8)	<0.001*	0.10 (0.023–0.46)	0.23	3.39 (0.45–25.32)
1,25(OH)_2_D deficiency (<18 pg/mL), *n* (%)	123 (73.2)	15 (88.2)	0.24	2.74 (0.60–12.47)		

Comorbidities, *n* (%)	Survivors (*n* = 168)	Non-survivors (*n* = 17)	Univariate analysis		Multivariate analysis	
			*p*-value	OR^§^ (95% CI)	*p*-value	OR^§^ (95% CI)
Diabetes mellitus	49 (29.3)	9 (52.9)	0.05*	2.70 (0.98–7.43)	0.46	1.66 (0.41–6.66)
Hypertension	85 (50.6)	7 (41.2)	0.45	0.68 (0.24–1.88)		
Coronary artery disease	47 (28.0)	2 (11.8)	0.24	0.34 (0.076–1.55)		
COPD	26 (15.5)	2 (11.8)	1.00	0.72 (0.15–3.37)		
Malignancy	10 (6.0)	2 (11.8)	0.30	2.10 (0.42–10.51)		

Laboratory findings, median (IQR)	Survivors (*n* = 168)	Non-survivors (*n* = 17)	Univariate analysis		Multivariate analysis	
			*p*-value	OR^§^ (95% CI)	*p*-value	OR^§^ (95% CI)
Hemoglobin (g/dL)	12.3 (10.3–13.5)	10.9 (8.5–12.5)	0.03*		0.99	1 (0.60–1.65)
Hematocrit (%)	37.5 (32.4–40.6)	34.4 (26.1–38.95)	0.17			
WBC (×10^3^ μL)	7.29 (5.75–11.21)	10.18 (6.65–13.55)	0.10			
Lymphocyte (×10^3^ μL)	1.34 (0.93–1.85)	1.18 (0.81–1.55)	0.34			
Neutrophil (×10^3^ μL)	5.18 (3.57–8.44)	7.47 (3.97–9.83)	0.13			
Platelet (×10^3^ μL)	219 (165.5–290.7)	192 (131–339)	0.75			
AST (IU/L)	28 (20–45)	37 (25–89)	0.04*		0.08	1 (0.99–1.02)
ALT (IU/L)	22 (13–37)	22 (15–34)	0.55			
Urea (mg/dL)	33.5 (25–42)	55 (35–133.5)	<0.001*		0.74	0.99 (0.98–1)
Creatinine (mg/dL)	0.84 (0.65–1.06)	1.07 (0.76–3.50)	0.01*			
LDH (U/L)	275 (209–345)	366 (247.5–537)	0.005*		0.36	0.99 (0.99–1)
Albumin (g/L)	35.8 (32–39.47)	32 (27.4–36.2)	0.008*		0.85	1.01 (0.87–1.17)
Ferritin (μg/L)	149.3 (54–409)	325 (158.95–645.2)	0.012*		0.34	1 (1–1.001)
Triglycerides (mg/dl)	113 (82.5–172)	133 (80–179)	0.483			
CK (U/L)	82 (48–145)	70.5 (48.25–351.7)	0.797			
Procalcitonin (ng/mL)	0.1 (0.005–0.23)	0.66 (0.16–3.02)	<0.001*		0.40	0.92 (0.77–1.10)
CRP (mg/L)	47.4 (12.8–119.7)	129 (20.5–185.5)	0.05*		0.60	0.99 (0.99–1)
Fibrinogen (mg/dL)	477 (401–551)	452 (376–594.5)	0.88			
PT (s)	13.3 (0–15.1)	15.3 (13.6–18.1)	0.003*			
APTT (s)	35.05 (31.72–38.60)	38.4 (34.9–48.25)	0.015*		0.009*	1.09 (1.02–1.16)
D-dimer (mg/L)	0.39 (0.17–0.91)	1.19 (0.42–2.29)	0.013*		0.77	1.06 (0.69–1.63)
Troponin (ng/I)	6 (3–13)	26 (9.5–50)	0.002*		0.003*	1 (1.003–1.005)
25(OH)D (ng/mL)	18.92 (12.79–25.47)	25.5 (20.71–31.15)	0.004*			
1,25(OH)_2_ D (pg/mL)	14.04 (10.42–18.30)	12.71 (8.53–14.88)	0.068			

SD, standard deviation; IQR, interquartile range; OR, odds ratio; CI, confidence interval; ICU, intensive care unit; COPD, chronic obstructive pulmonary disease; WBC, White blood cell; AST, aspartate aminotransferase; ALT, alanine aminotransferase; LDH, lactate dehydrogenase; CK, creatine kinase; CRP, C-reactive protein; PT, prothrombin time; APTT, activated partial thromboplastin time. ^§^ORs are presented according to the reference categories defined in the regression model. ^*^Statistically significant values.

## 4 Discussion

In our study, we evaluated the relationship between serum 25(OH)D and 1,25(OH)_2_D levels and the clinical severity of COVID-19, demonstrating that 1,25(OH)_2_D levels were significantly lower in patients with severe disease. The finding that 25(OH)D levels were lower in patients with moderate disease may hypothetically reflect increased conversion of 25(OH)D to its active metabolite, 1,25(OH)_2_D, during the inflammatory response. This supports the notion that the active form, 1,25(OH)_2_D, plays a more central role in modulating immune responses throughout the disease course. Differences in sampling timing, acute inflammatory status, or dynamic alterations in vitamin D metabolism during hospitalization may also have contributed to this unexpected observation. Our results are consistent with previous studies reporting that vitamin D deficiency is associated with adverse clinical outcomes in COVID-19 patients (21, 22, 26–28).

The literature regarding the association between low vitamin D levels and the risk of SARS-CoV-2 infection or COVID-19 related mortality remains inconsistent. While several studies have failed to demonstrate a significant relationship (10, 11, 29), many randomized and observational studies have reported that vitamin D deficiency is associated with a more severe disease course (17). In particular, 25(OH)D levels below 30 ng/mL have been linked to an increased likelihood of hospitalization, and vitamin D supplementation has been reported to reduce mortality in some cohorts (30). Numerous studies have also highlighted the influence of factors such as age, obesity, comorbidities, and sex on COVID-19 severity (31–33).

With respect to mortality, our findings revealed that neither age nor sex independently predicted death, whereas diabetes mellitus emerged as a significant risk factor. This aligns with previous studies demonstrating an increased mortality risk in COVID-19 patients with diabetes (15, 34, 35). Independent factors associated with severe disease identified in our study included prolonged hospitalization prior ICU admission, and low 1,25(OH)_2_D levels. The severe disease group exhibited longer hospital stays, a higher rate of ICU admission, and increased mortality. These findings are consistent with reports indicating that decreased 1,25(OH)_2_D levels correlate with greater risk of death, prolonged mechanical ventilation, and worse acute physiology and chronic health evaluation scores (16, 21). Previous studies conducted in patients with sepsis and critical illness have similarly demonstrated reduced circulating 1,25(OH)_2_D levels in association with adverse clinical outcomes, organ dysfunction, prolonged mechanical ventilation, and increased mortality. Similarly, reduced circulating 1,25(OH)_2_D levels have been associated with mortality in patients with sepsis (36, 37). These findings support the concept that dysregulated vitamin D metabolism may occur as part of the systemic inflammatory response and critical illness-related metabolic alterations. In this context, reduced 1,25(OH)_2_D levels observed in severe COVID-19 may reflect not only impaired vitamin D homeostasis but also the broader effects of inflammatory dysregulation and multiorgan dysfunction.

In severe COVID-19, laboratory abnormalities such as elevated AST, urea, creatinine, LDH, CRP, ferritin, procalcitonin, troponin, and PT, along with low albumin levels and lymphopenia, reflect the systemic inflammatory response to the disease. Previous studies have shown negative correlations between CRP and other inflammatory markers and circulating 25(OH)D levels (38). Beyond its role in calcium metabolism, vitamin D is a key regulator of immune responses and inflammation. Several studies have suggested that vitamin D deficiency may exacerbate the severity of SARS-CoV-2 infection (39, 40). Serum 25(OH)D concentrations above 30 ng/mL have been associated with general health benefits, while levels above 50 ng/mL may reduce the risk of viral infections, sepsis, and autoimmunity (40).

Although 1,25(OH)_2_D deficiency was significantly associated with severe COVID-19 in our study, overall vitamin D deficiency was not found to be independently associated with mortality. Multivariate analyses identified prior ICU admission, high troponin levels and prolonged APTT were independently associated with mortality. Although vitamin D deficiency was significantly associated with mortality in univariate analysis, this association did not persist after multivariate adjustment. This finding suggests that the relationship between vitamin D deficiency and mortality may be mediated or confounded by factors reflecting disease severity and clinical deterioration, such as ICU admission and critical illness-related complications. Nevertheless, the strong association between low 1,25(OH)_2_D levels and disease severity suggests that this active metabolite may serve as a valuable prognostic biomarker in clinical monitoring.

One possible explanation for the paradoxically lower 25(OH)D levels observed in the moderate group may be an increased peripheral conversion of 25(OH)D to the biologically active metabolite 1,25(OH)_2_D during the inflammatory response. Previous studies have demonstrated that COVID-19 may induce substantial alterations in vitamin D metabolism, including dysregulation of hydroxylation pathways and changes in extrarenal 1α-hydroxylase activity. In severe disease, systemic inflammation, cytokine-mediated metabolic dysfunction, and critical illness-related organ impairment may contribute to reduced circulating 1,25(OH)_2_D levels despite relatively preserved 25(OH)D concentrations. This observation suggests that active vitamin D metabolism, rather than total vitamin D stores alone, may play a more relevant role in the immunopathogenesis and clinical severity of COVID-19 (41).

In conclusion, our findings indicate that 1,25(OH)_2_D levels may be a more potential biomarker of COVID-19 severity than 25(OH)D levels. Therefore, assessment of 1,25(OH)_2_D concentrations may support clinical decision-making, particularly in patients with ARDS or those at risk of developing severe disease.

### 4.1 Limitations

This study has several limitations. First, its single-center, observational design limits the generalizability of the findings. Due to the retrospective design, certain pre-analytical variables (e.g., storage duration and processing variability) could not be fully standardized, which may have affected the measurements. In addition, key inflammatory markers such as interleukin-6 (IL-6) were not measured in this study. This limitation precludes a more comprehensive assessment of the relationship between vitamin D metabolites and systemic inflammatory response. Potential confounding factors that may influence vitamin D levels could not be fully assessed. The use of a single analytical method for vitamin D measurements may also introduce methodological limitations. Another limitation of this study is the absence of a healthy control group. During the COVID-19 pandemic, all clinical departments in our hospital were reorganized as inpatient pandemic wards, and hospitalized patients mainly consisted of moderate-to-severe COVID-19 cases. Therefore, inclusion of a healthy control group was not feasible under the pandemic conditions. Consequently, it remains possible that the observed 25-OHD deficiency reflects the high prevalence of vitamin D deficiency in the general population rather than being specific to COVID-19 infection. In addition, due to the short half-life of 1,25(OH)_2_D, the measured levels may not fully reflect the dynamic metabolic changes occurring throughout the course of the disease. Finally, the relatively limited sample size may have reduced the statistical power of the mortality analyses, potentially affecting the detection of some associations. Because the number of mortality events was limited, the statistical power of multivariate mortality analyses may have been reduced.

## 5 Conclusion

In our study, prolonged hospital stay, prior intensive care unit (ICU) admission, and reduced 1,25(OH)_2_D levels were found independently associated with severe COVID-19. ICU admission history, high troponin levels and prolonged APTT emerged as independent factors associated with mortality. Although our findings indicate that vitamin D deficiency was not independently associated with mortality, the observed association between low 1,25(OH)_2_D levels and greater disease severity suggests a potential clinical relevance of active vitamin D metabolism in severe COVID-19. Overall, our findings support the possible role of circulating 1,25(OH)_2_D as a candidate biomarker associated with disease severity in COVID-19 and similar infectious conditions, particularly in patients at risk for severe outcomes or ARDS.

## Data Availability

The datasets generated during the current study are available from the corresponding author upon reasonable request and in accordance with applicable data protection regulations. Requests to access the datasets should be directed to Esra Canbolat Unlu, esracanbolat@gmail.com.
